# Case-Control Study of Nodding Syndrome in Acholiland: Urinary Multi-Mycotoxin Screening

**DOI:** 10.3390/toxins13050313

**Published:** 2021-04-27

**Authors:** Jennifer Duringer, Rajarshi Mazumder, Valerie Palmer, A. Morrie Craig, Peter Spencer

**Affiliations:** 1 Department of Environmental & Molecular Toxicology, Oregon State University, Corvallis, OR 97331, USA; Jennifer.Duringer@oregonstate.edu; 2 Department of Neurology, David Geffen School of Medicine, University of California Los Angeles, Los Angeles, CA 90095, USA; R.Mazumder@mednet.ucla.edu; 3 Department of Neurology, Oregon Health & Science University, Portland, OR 97239, USA; palmerv@ohsu.edu; 4 Department of Biomedical Sciences, College of Veterinary Medicine, Oregon State University, Corvallis, OR 97331, USA; morriecraig@comcast.net

**Keywords:** Nodding Syndrome, mycotoxins, urine, Uganda

## Abstract

This case-control study adds to the growing body of knowledge on the medical, nutritional, and environmental factors associated with Nodding Syndrome (NS), a seizure disorder of children and adolescents in northern Uganda. Past research described a significant association between NS and prior history of measles infection, dependence on emergency food and, at head nodding onset, subsistence on moldy maize, which has the potential to harbor mycotoxins. We used LC-MS/MS to screen for current mycotoxin loads by evaluating nine analytes in urine samples from age-and-gender matched NS cases (n = 50) and Community Controls (CC, n = 50). The presence of the three mycotoxins identified in the screening was not significantly different between the two groups, so samples were combined to generate an overall view of exposure in this community during the study. Compared against subsequently run standards, α-zearalenol (43 ± 103 µg/L in 15 samples > limit of quantitation (LOQ); 0 (0/359) µg/L), T-2 toxin (39 ± 81 µg/L in 72 samples > LOQ; 0 (0/425) µg/L) and aflatoxin M1 (4 ± 10 µg/L in 15 samples > LOQ; 0 (0/45) µg/L) were detected and calculated as the average concentration ± SD; median (min/max). Ninety-five percent of the samples had at least one urinary mycotoxin; 87% were positive for two of the three compounds detected. While mycotoxin loads at NS onset years ago are and will remain unknown, this study showed that children with and without NS currently harbor foodborne mycotoxins, including those associated with maize.

## 1. Introduction

Nodding Syndrome (NS) is a brain disorder (tauopathy) of childhood and adolescence that has been present over the past 20–25 years in epidemic form in northern Uganda and South Sudan [1,2,3,4,5,6]. Internal displacement, severe food shortages, vermiform infection, and lack of medicine and immunization are common to NS-affected populations in these areas. Children with this disease are born normally and undergo unremarkable perinatal development before growth slows and the child becomes physically and mentally stunted, coincident with behavioral changes, periodic head nodding, and convulsions. The short stature and developmental delay seen in some children with NS and its phenotypic variant, Nakalanga syndrome, raise the possibility of neuroendocrine dysfunction [7,8].

In 2001–2002, a team assembled by the World Health Organization (WHO) to investigate an outbreak of NS in then-southern Sudan noted a strong association between head nodding and ingestion of food (serena, a red sorghum) delivered by the World Food Programme [5]. Plants with direct or indirect neurotoxic potential (ackee, cassava, grasspea, sorghum, vetch, and yams) were ruled out as risk factors, but the possibility that head nodding was associated with food contaminated with pre-harvest or post-harvest mycotoxins (secondary mold metabolites) could not be excluded [5,9]. In northern Uganda (Acholiland), an epidemic of NS (1997–2015) was associated with civil war-related movement of the population to internal displacement camps, where conditions were squalid, infectious disease rampant, and food quality very poor [10]. In March 2014, a case-control study of medical, nutritional, and other risk factors associated with NS among 5–18 year-old Acholi children found a significant case association with reports of prior measles infection; higher monthly onset of NS when food stocks were low; greater use of emergency food delivered by the World Food Programme; and food dependence on moldy plant food, in particular moldy maize, at onset of head nodding [11]. Moldy maize consumed in Africa is frequently contaminated with mycotoxins from the following groups: aflatoxins, fumonisins, trichothecenes, and zearalenones [12,13,14,15,16].

The degree to which mycotoxins as a whole influence global health is not yet fully understood and appreciated, a sentiment echoed by others [17,18]. Simply establishing baseline data of exposure in susceptible populations remains to be done in many regions of the globe. Epidemiological data on foodborne diseases are lacking, particularly in low-income countries, and outbreaks often go unrecognized and unreported [19]. Populations most at risk have limited regulatory oversight or prevention measures to avoid consumption of contaminated food [20], as many rely on subsistence farming and local, unregulated markets [21]. As surveillance studies are completed and the relative burden of mycotoxins comes to light in a given region, this information must be utilized to develop a concerted response among all stakeholders, from producers to consumers, with an emphasis on integration of local voices and infrastructure. Further, the authors stressed that regulation and surveillance are important but that sensitive, cost-effective methods of detection are needed for enforcement of mycotoxin limits under a food safety program.

Relevant biomarkers of mycotoxin exposure with supporting validation and implementation in human subjects across four continents were recently reviewed [22,23,24]. For the mycotoxins of concern in moldy maize, candidate biomarkers include urinary aflatoxin M1, sphinganine:sphingosine ratio for fumonsins, deoxynivalenol, and zearalenone/α-zearalenol/β-zearalenol. In the current study, we examined mycotoxin biomarkers in the urine of Acholi children both with and without NS (n = 50/group). Mycotoxin load reflects exposure to fungal products during the preceding few days. As such, this study aimed to demonstrate that, rather, beyond NS, mycotoxin contamination of food and feed in Africa is a major problem with implications that affect human and animal health and the economy [16,25,26]. Multi-mycotoxin biomonitoring surveys such as this add to the growing database of direct exposure estimates, which will aid in identifying populations vulnerable to development of mycotoxicoses and elucidate the possible contribution mycotoxins make to exacerbating morbidity of regional diseases. This information can provide a framework upon which better, more timely risk assessment and mitigation strategies can be developed for these fungal contaminants.

## 2. Results

Table 1 identifies the mycotoxins detected in the 50 NS and 50 Community Control (CC) samples that were analyzed using a multi-mycotoxin method, which surveyed for nine mycotoxins and their metabolites. Results should be viewed semi-quantitatively, as samples were compared against subsequently run matrix-matched standards. α-Zearalenol was detected in 93 of the 100 samples; of those, only 15 exceeded the limit of quantitation (LOQ), with mean concentration ± standard deviation (SD) of 43 ± 103 µg/L. Other mycotoxins detected in this survey were T-2 (86/100 samples, with 72 above the LOQ and an average concentration of 39 ± 81 µg/L) and aflatoxin M-1 (48/100 samples, with 15 above the LOQ and an average concentration of 4 ± 10 µg/L). NS and CC samples were not significantly different from each other for any of these mycotoxins (*p* > 0.05). Signals for the quantitative and qualitative fragments of zearalenone-14-O-glucuronide were detected, but no standard was commercially available; thus, levels could not be quantified in the 73/100 samples in which it was identified.

Ninety-five of the samples were positive for at least one mycotoxin for the 100 Acholi children evaluated, with 87 samples being positive for two of the three quantitatively determined mycotoxins. The remaining mycotoxins in our screening assay (ZEN, β-ZEL, HT-2, FB_1_, FB_2_, and OTA) were not detected in the urine samples above their LOQ. Correlation was run comparing the three mycotoxins to each other in all samples, and none achieved a value of correlation above 0.03, indicating that, within an individual, there was no correlation in mycotoxin concentration for the three mycotoxins detected.

## 3. Discussion

We subjected urine samples from NS and CC children to multi-biomarker analysis for exposure to mycotoxins, which generally reflects recent (days) dietary intake [27]. Since all children at the time of sampling were living in villages under peacetime conditions, instead of the privation and malnutrition that typified their earlier life in IDP camps, no significant differences in mycotoxin content were expected or revealed (*p* > 0.05). However, we found evidence among the 100 samples children that their current (2014) diet exposed them to a range of mycotoxins with immunotoxic, neurotoxic, and genotoxic properties. Another study of this population found high levels of total aflatoxin and ochratoxin in mostly millet, sorghum, maize, and groundnuts in households both with and without children with NS [28]. Similarly to the present study, no significant association was found between dietary mycotoxins and incidence of NS. A limitation of their study also included sampling many years (2014–2015) after the temporal peak of diagnosed NS cases from Ugandan households. However, together, these results demonstrate the need to analyze factors that promote food spoilage in this rural Ugandan community, with the goal of improving food quality to reduce exposure to hazardous mycotoxins as a public health measure.

### 3.1. Mycotoxins Detected and Their Impacts on Nutrition, Growth, and Child Health

Mycotoxins have more recently been targeted for their effects on nutrition and immunity, particularly as they affect growth and development in infants and children [16,19,20,21,26,29,30,31,32,33]. Stunting (child’s height is two standard deviations or more below the WHO growth reference [20]) and being underweight are manifestations exacerbated by malnutrition in infants and children who are already at a higher risk for the effects of mycotoxin exposure due to their higher rate of food intake per kg body weight [34]. The International Agency for Research on Cancer’s Working Group report on mycotoxin exposure in low and middle-income countries provides a comprehensive review on the subject [31]. Their analysis concluded that mycotoxin exposure contributes to child growth impairment independent of, and together with, other risk factors that may cause stunting. Exposure to mycotoxins may thus have played a role in the stunted growth of NS versus CC children [9]. Aflatoxins, trichothecenes and fumonisins have been the predominant compounds discussed thus far in the literature, but the mechanism of toxicity by which these and other mycotoxins contribute to infant and young child growth impairment need to be further developed, especially in light of the fact that individuals are often chronically exposed to a multitude of compounds in the diet [32,33,35,36]. For example, a study in Tanzania of 6–14 month-old children found urinary fumonisin B_1_ (by itself or in combination with aflatoxin exposure) to be negatively associated with child growth [37]. Fumonisins inhibit ceramide synthase, thus altering sphingolipid metabolism and thereby disrupting the function of membrane proteins such as the folate-transporter proteins (folate receptor α in humans) [38]. Inhibition of folate transport can cause neural tube, craniofacial, and other developmental defects, which have been noted in populations with a history of fumonisin consumption [38,39].

The Sanitation Hygiene Infant Nutrition Efficacy (SHINE) trial in Zimbabwe hypothesized that mycotoxin exposure contributes to child stunting through environmental enteric dysfunction and disruption of the insulin-like growth factor 1 axis [40], pointing to altered nutrient absorption/intestinal function and immunomodulation as possible mechanisms. In another study, average birthweight did not differ among NS cases and controls, but mean body weight at time of examination was significantly lower for NS cases [11]. It is plausible that exposure to immunosuppressive fungal metabolites may set up the children to be more susceptible to secondary conditions, such as infestation with the nematodes *Mansonella perstans* and *Onchocerca volvulus* [41], a risk factor for NS [2,42]. In terms of in utero exposure, studies have demonstrated the transmissibility of mycotoxins across the maternal-fetal barrier [32,43,44,45,46,47,48]. Mycotoxin presence in breast milk has been reviewed and a summary made of the aflatoxins, ochratoxin A, zearalenone, and fumonisin B1 found to date in that matrix [49]. The authors stressed that we have much to learn about the breadth and actual contamination level of these compounds given recent advances in instrumentation and multi-compound detection methodologies. Further, rate of lactational transfer and the role of biotransformation, from both the mother and infant, will be important in making an accurate risk assessment for exposed infants.

As a whole, no major differences were detected in the 100 Acholi children evaluated, either for an individual or group of mycotoxins in the biomatrix assessed (Table 1). Ninety-five of the urine samples were positive for at least one mycotoxin, with 87 samples being positive for two of the three mycotoxins quantitated. α-Zearalenol was found in 15 individuals from both the NS and CC groups (Table 1). The mean concentration for the NS group was 38 ± 97 µg/L, while that for the CC group was 52 ± 112 µg/L. This difference was deemed not significant (*t*-test, *p* = 0.54). Zearalenone-4-*O*-glucuronide was also found in the majority of samples (73/100); combined with α-zearalenol, these results indicate exposure to zearalenone as the most significant contaminating mycotoxin in the current food supply of the children evaluated. Zearalenone is a non-steroidal estrogenic compound produced mainly by *Fusarium* spp. on grains and is associated with reproductive disorders such as decreased conception, abortion, and hyperestrogenism, but may also be developmentally immunotoxic [50].

Aflatoxins (B_1_, B_2_, G_1_, and G_2_) are produced by *Aspergillus* spp. on peanuts, maize, and other grains common in African diets; they primarily affect the liver, causing hepatic necrosis leading to cirrhosis and cancer if ingested in large enough quantities (acute aflatoxicosis) or chronically consumed over a longer period of time, especially with co-exposure to the hepatitis B virus [51,52]. In addition, aflatoxins have immunosuppressive effects and, due to their ability to bind with DNA, affect protein synthesis [51], an absolute requirement for growth and development. The presence of metabolite aflatoxin M1 in the urine samples from the current study (4 ± 10 µg/L of all children (Table 1)) reflects exposure to aflatoxin B1. Echodu and co-authors [28] also found high levels of aflatoxin in foods consumed from Ugandan communities both with and without NS cases. The authors suggest how, despite there being no significant correlation with NS, chronic aflatoxin exposure could act as a cofactor in NS disease development or participate in generally overwhelming normal developmental processes in children, as found in a recent review on adverse reproductive outcomes in African children exposed to mycotoxins [32].

Trichothecenes (most notably deoxynivalenol (DON), nivalenol, T-2 toxin, HT-2 toxin, and diacetoxyscirpenol) obstruct protein biosynthesis via inhibition of peptidyl transferase; they are found mainly in cereals such as wheat, maize, and barley, and less frequently in sorghum and rice [34,51]. Trichothecenes also disrupt gastrointestinal function and interfere with growth hormone activity. Chronic exposure of animals resulted in decreased food intake and weight gain, altered immune function, and negative developmental sequelae [20,53]. T-2 toxin was frequently detected in samples from the current study (72% of samples), with an average of 39 ± 81 µg/L in all children (Table 1).

### 3.2. Dietary Exposure to Mycotoxins in African Food Plants

The presence of mycotoxins in the urine reflects food intake of mold-bearing plant materials while subjects were living at home in a time of peace without food shortage. It is reasonable to posit that the mycotoxin load was greater (delayed harvest, poor storage, damp conditions [26]) when these children were subjected to severe food shortages that required them to eat moldy plant components, notably moldy maize, during their wartime internment [11]. An example of similar circumstances was found with retrospective association of T-2 toxin and HT-2 toxin in moldy grains consumed in eastern Siberia at times over the last 100 years and the development of alimentary toxic aleukia in that location [54]. As proof of principle, samples of sorghum (*Sorghum bicolor*) were collected for mycotoxin analysis from a field proximate to an Acholi family with a NS child [55]. Seed samples were classified as “old” (long past harvest, n = 4), “young” (n = 4), and “neither old nor young” (n = 5) by a knowledgeable local community member. It was of interest to analyze samples taken from different harvests because displacement of communities in this region often forced them to scavenge and consume food of questionable quality. Beauvericin was the most prominent mycotoxin detected, at an average concentration of 552 µg/kg in the “old” samples; “young” and “neither old nor young” contained 251 and 141 µg/kg, respectively. Beauvericin is an apolar lipophilic cyclohexadepsipeptide that forms ionophores in plasma membranes. Its ability to inhibit L-type voltage-dependent Ca^2+^ channels in a neuronal cell line suggests the possibility that it may increase sensitivity to kainate-induced neurotoxicity (seizures and neuronal damage) [56]. In addition, emodin, alternariol methyl ether, alternariol, zearalenone, deoxynivalenol, aflatoxin G2, agroclavine, festuclavine, and dihydroergosine were identified in at least one of the 13 samples. However, a variety of dietary factors examined in another study found edible smut fungus to be inversely linked to NS (non-significant, *p* = 0.065) [11], suggesting that mycotoxins on their own cannot be implicated in disease etiology. 

In addition to the toxic effects of individual mycotoxins, co-exposure to numerous compounds at once is likely to be common given the observance of multiple mycotoxins in feed/food surveys done across the globe [32]. This needs to be considered in safety and toxicological evaluations, as a given group of compounds could enhance or reduce the overall toxicity to the organism that consumes them. The potent Group 1 carcinogen aflatoxin B_1_, as well as other members of the aflatoxin and fumonisin families, have been detected with high frequency in African maize in addition to deoxynivalenols and zearalenones; when analysis methodologies allowed, ochratoxin A, moniliformin, 3-nitropropionic acid, cyclopiazonic acid, citrinin, roquefortine C, sterigmatocystin, beauvericin, and the enniatins were variably identified as well [16,57,58,59,60,61,62]. Ochratoxin A occurs in a wide range of human foods including cereals, coffee, wine, beans, nuts, and meat and milk products from animals consuming contaminated feed [34,51]. It is hepatoxic, immunosuppressive, teratogenic, and a group 2B possible human carcinogen [63]. Also known as vomitoxin for its emetic effect on animals, DON can cause general gastrointestinal distress, weight loss, and feed refusal/anorexic behaviors. DON is one of the most common mycotoxins assayed for and detected in urinary surveys irrespective of geographic location, as seen in both African [58,61,64] and European [27,65,66] studies. Aflatoxin, ochratoxin, and DON were all detected in a home survey of stored grains comparing households with and without NS children; no significant difference was found between the two groups in the total average concentration detected [28].

Maize-based porridge samples [12] and maize kernels [13] sampled from Tanzanian villages showed severe contamination with fumonisins and aflatoxins, while zearalenone and DON were also detected. Aflatoxins, fumonisins, trichothecenes, and beauvericin predominated in maize assessed from Burkina Faso and Mozambique [14]. The Republic of Benin analyzed local maize flour and found fumonisins and beauvericin to be present [15]. In Uganda, Sudan, and Tanzania, aflatoxins in millet, sorghum flour and nut products, and aflatoxins and fumonisins in maize, were listed previously as significant detects [16]. PCR analysis of mold species on Tunisian and Egyptian sorghum determined that *Fusarium incarnatum*, *Aspergillus flavus*, and *A. niger* were the main mycoflora present [67]; isolates were able to produce aflatoxins, ochratoxin A, and zearalenone. Sorghum, an imported red variety that was statistically associated with NS in South Sudan [9] but not in Uganda [2,11], is prone to spoilage by *Fusarium verticillioides*; this fungus elaborates fumonisin B1, which blocks sphingolipid synthesis and causes leukoencephalomalacia in horses and hippocampal lesions in laboratory species [68].

Less commonly reported mycotoxins may also be present in food and feed originating in Africa. Dihydrorergosine is the principal toxic alkaloid produced by sorghum ergot (*Claviceps africana*) [69] and has been associated with agalactia, suppressed prolactin, feed refusal, and hyperthermia in livestock [70,71]. Sulochrin is produced by *Aspergillus* and *Penicillium* spp. and inhibits eosinophil functions including degranulation [72,73]. Emodin is an anthraquinone produced by *Aspergillus* spp. and other fungal species, contaminating many plants used in herbal medicine in addition to those used as [74]. It has a number of therapeutic properties but can also be hepatotoxic and nephrotoxic and affect reproduction [75]. Emodin was detected previously in maize and feed from Burkina Faso and Mozambique [14]; in that study, the authors emphasized the need to screen for non-regulated compounds such as emodin to maintain a comprehensive picture of mycotoxin exposure in a given environment.

### 3.3. Study Limitations

While we utilized LC-MS/MS in the current study, a number of technical approaches could improve sensitivity and therefore detection of mycotoxins, including use of a more advanced triple quadrupole or time-of-flight mass spectrometer capable of lower detection and quantification limits. In addition, the sample preparation method of “dilute and shoot” has speed and cost advantages along with an ability to recover a chemically diverse array of compounds and their conjugates; however, it was optimized to capture high to moderate concentrations rather than low or background levels [76]. Moreover, sample values here must be considered semi-quantitative as they were compared against subsequently run standards. More advanced methods for mycotoxin exposure biomarkers in urine have been developed since our samples were evaluated; these cleave mycotoxins by glucuronidase incubation, followed by sample clean-up using solid-phase extraction columns and ^13^C- or deuterated internal standards [77]. This acknowledges the presence of Phase II conjugates in the urine that have likely escaped detection in methods that analyze only for the parent mycotoxin, thereby underestimating exposure to metabolites with potential toxicity [78].

## 4. Conclusions

We carried out a multi-mycotoxin screen for nine analytes in the urine of children with Nodding Syndrome (n = 50) and healthy Community Controls (n = 50) residing in a remote Acholi village of northern Uganda. Mycotoxins were detected in the urine, including α-zearalenone, aflatoxin M1, and T-2 toxin, with no significant difference between the two groups. However, because the samples were collected years after onset of NS, a role for mycotoxins in the etiology of NS or associated nematode infection cannot be ruled out specifically as secondary factors that contribute to a generalized decrease in immune and/or growth function. Mounting evidence that food-derived mycotoxins play a role in altering optimal development in African children and the frequency of their detection in this population call for further investigation with optimized assays to detect mycotoxins, identify their sources, and minimize exposure by avoidance of contaminated food.

## 5. Materials and Methods

### 5.1. Setting

The study was set in the NS epicenter in northern Uganda. Between 1987 and 2006/2008, this region was a civil war zone involving the Lord’s Resistance Army (LRA), the Sudan People’s Liberation Army, and the Ugandan People’s Defence Force. Beginning in 1996, Acholi civilians were moved into internally displaced peoples (IDP) camps guarded by Ugandan government forces. In these camps, there was social breakdown, rising incidence of mental health disorders, HIV/AIDs, alcoholism, malnutrition, and eventual reliance on food aid. They became breeding grounds for malnutrition and deaths resulting from cholera, measles, and other preventable diseases. In 2008, following the expulsion of the LRA, the Acholi began to return to village homelands and rebuild their society. After recognition of NS in Kitgum District in 2008, the Ugandan government registered children, established a government screening and treatment center, and distributed anticonvulsants and nutritional supplementation for affected children.

### 5.2. Study Approval and Informed Consent

Study and instrument design were approved by the Institutional Review Boards of Oregon Health & Science University (Portland, OR, USA) and Oregon State University (Corvallis, OR, USA), in concert with the Research and Ethics Committee of the School of Health Sciences of Makarere University (Kampala, Uganda). The study was approved by the Uganda National Council for Science and Technology, the Office of the President of Uganda, and Chairman, Local Council III, Kitgum District, Uganda. Informed consent was obtained for the collection of urine for large-scale mass spectrometric analysis of mycotoxin content. Approval code: IRB00008470, Approval date 1 November 2013–14 September 2021. Caretakers of children were paid the equivalent of U.S. $10.00 in local currency to compensate for their time. One half of each subject’s biological fluids was banked with the Department of Food Technology and Nutrition, College of Agricultural and Environmental Sciences, Makerere University, Kampala. Permission to transport the other half of each subject’s biological fluids to the USA for analysis was granted by the Uganda National Council for Science and Technology.

### 5.3. Urine Sample Collection

Families with and without children with a NS diagnosis were requested by the Village Health Team Leader to visit the designated research study site. The medical status of each child was checked against a list of all NS subjects that recorded the month and year of onset of head nodding. The case-control study enrolled 50 children aged 5–18 years with NS and an equivalent number of seizure-free children from the community matched for age (50/50) and gender (42/50) as Community Controls. Each child gave one sample of free-caught urine in a cup (10–20 mL) during daylight hours, which was immediately placed on ice.

Urine samples were transported frozen by car from Kitgum to Kampala, then separated into two sets. One half of the biological fluids was retained in Uganda, while the balance was transported in a Yeti Tundra Cooler on passenger flights from Entebbe, Uganda to Portland, Oregon. Samples were stored at −20 °C prior to mycotoxin analysis.

### 5.4. Chemicals

Acetonitrile (ACN) and methanol were of LC-MS grade (EMD Millipore, Billerica MA USA); acetic acid (LC-MS grade) was purchased from Sigma Aldrich (St. Louis, MO USA). Ultrapure water (18 MΩ cm^−1^) was obtained from a PURELAB Ultra Genetic system (Elga, Marlow, Buckinghamshire, UK). Zearalenone (ZEN), α-zearalenol (α-ZEL), β-zearalenol (β-ZEL), T-2 toxin (T-2), and HT-2 toxin (HT-2) standards came dissolved in ACN, while fumonisin B_1_ (FB_1_) and fumonisin B_2_ (FB_2_) standards were dissolved in 50:50 water:ACN; all were purchased from Romer Labs (Getzersdorf, Austria). Aflatoxin M1 (AFM1) and ochratoxin A (OTA) (Enzo Life Sciences, Farmingdale, NY, USA) were dissolved after arrival in ACN for dilution and generation of standard curves.

### 5.5. Sample Preparation-Urine Extraction for Mycotoxins

Urine was prepared using a dilute-and-shoot approach [79]. Urine was removed from the freezer, allowed to reach room temperature, and then centrifuged for 3 min at 10,000 rpm (Eppendorf (Hamburg, Germany)). Supernatant (100 µL) was removed and added to 900 µL of a 10:90 (*v*/*v*) ACN:water mixture; the sample was vortexed, then sealed for analysis by LC-MS/MS.

### 5.6. LC-MS/MS Analysis

Coded samples were subjected to a multi-mycotoxin LC-MS/MS screening assay for 9 mycotoxins (ZEN, α-ZEL, β-ZEL, T-2, HT-2, FB_1_, FB_2_, AFM1, and OTA) on an ABI/SCIEX 3200 QTRAP LC-MS/MS system (Applied Biosystems, Foster City, CA, USA) via electrospray ionization, with separation performed using a Perkin Elmer (Waltham, MA, USA) Series 200 HPLC connected to an Atlantis T3 column (150 × 3.0 mm, Waters (Milford, MA, USA)) with a 4 × 3 mm security guard cartridge of similar packing [79]. The methodology used was chosen on the basis of available instrumentation and ability to quantify the major mycotoxins of concern that are globally monitored in the human food supply.

Mobile phases consisted of water (A) or ACN (B), each with 0.1% acetic acid, which were run in a gradient program at 600 µL/min. Each sample was run in triplicate; each replicate had two injections taken, one each for both positive and negative modes, which captured 9 mycotoxins and mycotoxin metabolites as described in Warth et al. [79] via scheduled (180 s window) multiple reaction monitoring (MRM) of two transitions (quantitative and qualitative) per compound. Dilution solvent (1:9 ACN:H_2_O) served as the blank sample.

### 5.7. Data Analysis

Qualitative analyses were conducted blind to the group status of each child’s specimen using Analyst 1.6.2, MultiQuant 3.0.1 (Applied Biosystems, Framingham, MA, USA) and Excel (Microsoft, Redmond, WA, USA). The presence of a mycotoxin was confirmed when the signal was equal to or greater than the limit of quantitation (LOQ, the concentration at which the analyte had a precision and accuracy that did not exceed greater than 20% of the coefficient of variation [80]), both quantitative and qualitative transitions were present, and retention time was at the expected time in the chromatogram. After samples were evaluated for the presence of mycotoxins, they were quantified against a standard curve with subsequently run standards (matrix-matched standards spiked into urine and diluted as described for samples above) developed in MultiQuant. The limit of detection (LOD) and LOQ for the mycotoxins were as follows: ZEN (10, 50 ng/mL), α-ZEL (20, 20 ng/mL), β-ZEL (25, 25 ng/mL), T-2 (2.5, 10 ng/mL), HT-2 (0.2, 5 ng/mL), FB_1_ (0.1, 2.5 ng/mL)_,_ FB_2_ (0.5, 2.5 ng/mL), AFM1 (2.5, 2.5 ng/mL), and OTA (0.5, 0.5 ng/mL) (Supplementary Table S1). See Supplementary Table S1 and Supplementary Figure S1 for additional method validation information. After mycotoxin analysis was complete, the code was broken and results compared among NS and CC subjects using a t-test with significance determined as *p* < 0.05.

## Figures and Tables

**Table 1:** Mycotoxins detected in screen of urine from Ugandan children with Nodding Syndrome and age-and gender-matched Community Controls.

	Nodding Syndrome (N = 50)			Community Controls (N = 50)			Samples Combined (N = 100)		
Mycotoxin	(%) Positive	Mean (µg/L) ^a^	Median (Min, Max) (µg/L)	(%) Positive	Mean (µg/L) ^a^	Median (Min, Max) (µg/L)	(%) Positive	Mean (µg/L) ^a^	Median (Min, Max) (µg/L)
α-Zearalenol	6 (12%)	34 ± 94	0 (0, 317)	9 (18%)	52 ± 112	0 (0, 359)	15 (15%)	43 ± 103	0 (0, 359)
T-2	35 (70%)	29 ± 66	0 (0, 288)	37 (74%)	49 ± 93	0 (0, 425)	72 (72%)	39 ± 81	0 (0, 425)
Aflatoxin M1	7 (14%)	4 ± 9	0 (0, 36)	8 (16%)	4 ± 10	0 (0, 45)	15 (15%)	4 ± 10	0 (0, 45)

^a^ Numerical values represent the mean of all samples ± standard deviation.

## Supplementary Materials

The following are available online at https://www.mdpi.com/article/10.3390/toxins13050313/s1, Table S1. Validation data and Figure S1. Extracted ion chromatograms of mycotoxins detected in urine of Acholi children, as compared to method blank (1:9 ACN:water).

## References

[1] Dowell S.F., Sejvar J.J., Riek L., Vandemaele K.A.H., Lamunu M., Kuesel A.C., Schmutzhard E., Matuja W., Bunga S., Foltz J. (2013). Nodding Syndrome. Emerg. Infect. Dis..

[2] Foltz J.L., Makumbi I., Sejvar J.J., Malimbo M., Ndyomugyenyi R., Atai-Omoruto A.D., Alexander L.N., Abang B., Melstrom P., Kakooza A.M. (2013). An Epidemiologic Investigation of Potential Risk Factors for Nodding Syndrome in Kitgum District, Uganda. PLoS ONE.

[3] Pollanen M.S., Onzivua S., Robertson J., McKeever P.M., Olawa F., Kitara D.L., Fong A. (2018). Nodding Syndrome in Uganda Is a Tauopathy. Acta Neuropathol..

[4] Spencer P., Palmer V., Jilek-Aall L. (2013). Nodding Syndrome: Origins and Natural History of a Longstanding Epileptic Disorder in Sub-Saharan Africa. Afr. Health Sci..

[5] Tumwine J., Vandemaele K., Chungong S., Richer M., Anker M., Ayana Y., Opoka M., Klaucke D., Quarello A., Spencer P. (2012). Clinical and Epidemiologic Characteristics of Nodding Syndrome in Mundri County, Southern Sudan. Afr. Health Sci..

[6] Olum S., Scolding P., Hardy C., Obol J., Scolding N.J. (2020). Nodding Syndrome: A Concise Review. Brain Commun..

[7] Föger K., Gora-Stahlberg G., Sejvar J., Ovuga E., Jilek-Aall L., Schmutzhard E., Kaiser C., Winkler A.S. (2017). Nakalanga Syndrome: Clinical Characteristics, Potential Causes, and Its Relationship with Recently Described Nodding Syndrome. PLoS Negl. Trop. Dis..

[8] Spencer P.S., Mazumder R., Palmer V.S., Pollanen M.S. (2019). Nodding Syndrome Phenotypes. Rev. Neurol..

[9] Spencer P., Vandemaele K., Richer M., Palmer V., Chungong S., Anker M., Ayana Y., Opoka M., Klaucke B., Quarello A. (2013). Nodding Syndrome in Mundri County, South Sudan: Environmental, Nutritional and Infectious Factors. Afr. Health Sci..

[10] Landis J.L., Palmer V.S., Spencer P.S. (2014). Nodding Syndrome in Kitgum District, Uganda: Association with Conflict and Internal Displacement. BMJ Open.

[11] Spencer P.S., Mazumder R., Palmer V.S., Lasarev M.R., Stadnik R.C., King P., Kabahenda M., Kitara D.L., Stadler D., McArdle B. (2016). Environmental, Dietary and Case-Control Study of Nodding Syndrome in Uganda: A Post-Measles Brain Disorder Triggered by Malnutrition?. J. Neurol. Sci..

[12] Geary P.A., Chen G., Kimanya M.E., Shirima C.P., Oplatowska-Stachowiak M., Elliott C.T., Routledge M.N., Gong Y.Y. (2016). Determination of Multi-Mycotoxin Occurrence in Maize Based Porridges from Selected Regions of Tanzania by Liquid Chromatography Tandem Mass Spectrometry (LC-MS/MS), a Longitudinal Study. Food Control.

[13] Kamala A., Ortiz J., Kimanya M., Haesaert G., Donoso S., Tiisekwa B., De Meulenaer B. (2015). Multiple Mycotoxin Co-Occurrence in Maize Grown in Three Agro-Ecological Zones of Tanzania. Food Control.

[14] Warth B., Parich A., Atehnkeng J., Bandyopadhyay R., Schuhmacher R., Sulyok M., Krska R. (2012). Quantitation of Mycotoxins in Food and Feed from Burkina Faso and Mozambique Using a Modern LC-MS/MS Multitoxin Method. J. Agric. Food Chem..

[15] Ediage E.N., Di Mavungu J.D., Monbaliu S., Van Peteghem C., De Saeger S. (2011). A Validated Multianalyte LC-MS/MS Method for Quantification of 25 Mycotoxins in Cassava Flour, Peanut Cake and Maize Samples. J. Agric. Food Chem..

[16] Darwish W.S., Ikenaka Y., Nakayama S.M.M., Ishizuka M. (2014). An Overview on Mycotoxin Contamination of Foods in Africa. J. Vet. Med. Sci..

[17] Brown G.D., Denning D.W., Gow N.A.R., Levitz S.M., Netea M.G., White T.C. (2012). Hidden Killers: Human Fungal Infections. Sci. Transl. Med..

[18] Denning D.W., Bromley M.J. (2015). How to Bolster the Antifungal Pipeline. Science.

[19] Havelaar A.H., Kirk M.D., Torgerson P.R., Gibb H.J., Hald T., Lake R.J., Praet N., Bellinger D.C., de Silva N.R., Gargouri N. (2015). World Health Organization Global Estimates and Regional Comparisons of the Burden of Foodborne Disease in 2010. PLoS Med..

[20] Wu F., Groopman J.D., Pestka J.J. (2014). Public Health Impacts of Foodborne Mycotoxins. Annu. Rev. Food Sci. Technol..

[21] Shephard G.S. (2008). Impact of Mycotoxins on Human Health in Developing Countries. Food Addit. Contam. Part A.

[22] Al-Jaal B.A., Jaganjac M., Barcaru A., Horvatovich P., Latiff A. (2019). Aflatoxin, Fumonisin, Ochratoxin, Zearalenone and Deoxynivalenol Biomarkers in Human Biological Fluids: A Systematic Literature Review, 2001–2018. Food Chem. Toxicol..

[23] Vidal A., Mengelers M., Yang S., Saeger S.D., Boevre M.D. (2018). Mycotoxin Biomarkers of Exposure: A Comprehensive Review. Compr. Rev. Food Sci. Food Saf..

[24] Ezekiel C.N., Warth B., Ogara I.M., Abia W.A., Ezekiel V.C., Atehnkeng J., Sulyok M., Turner P.C., Tayo G.O., Krska R. (2014). Mycotoxin Exposure in Rural Residents in Northern Nigeria: A Pilot Study Using Multi-Urinary Biomarkers. Environ. Int..

[25] Dutton M.F. (2009). The African Fusarium/Maize Disease. Mycotoxin Res..

[26] Wagacha J.M., Muthomi J.W. (2008). Mycotoxin Problem in Africa: Current Status, Implications to Food Safety and Health and Possible Management Strategies. Int. J. Food Microbiol..

[27] Heyndrickx E., Sioen I., Huybrechts B., Callebaut A., De Henauw S., De Saeger S. (2015). Human Biomonitoring of Multiple Mycotoxins in the Belgian Population: Results of the BIOMYCO Study. Environ. Int..

[28] Echodu R., Edema H., Malinga G.M., Hendy A., Colebunders R., Moriku Kaducu J., Ovuga E., Haesaert G. (2018). Is Nodding Syndrome in Northern Uganda Linked to Consumption of Mycotoxin Contaminated Food Grains?. BMC Res. Notes.

[29] Khlangwiset P., Shephard G.S., Wu F. (2011). Aflatoxins and Growth Impairment: A Review. Crit. Rev. Toxicol..

[30] Smith L.E., Stoltzfus R.J., Prendergast A. (2012). Food Chain Mycotoxin Exposure, Gut Health, and Impaired Growth: A Conceptual Framework. Adv. Nutr..

[31] Wild C.P., Miller J.D., Groopman J.D. (2015). Mycotoxin Control in Low- and Middle-Income Countries. International Agency for Research on Cancer. Lyon, France (IARC Working Group Reports, No. 9). https://www.ncbi.nlm.nih.gov/books/NBK350558/.

[32] Eze U.A., Routledge M.N., Okonofua F.E., Huntriss J., Gong Y.Y. (2018). Mycotoxin Exposure and Adverse Reproductive Health Outcomes in Africa: A Review. World Mycotoxin J..

[33] Tshalibe R.S., Rheeder J.P., Alberts J.F., Taljaard-Krugell C., Gelderblom W.C.A., Shephard G.S., Lombard M.J., Burger H.-M. (2020). Multi-Mycotoxin Exposure of Children (0–24 Months) in Rural Maize-Subsistence Farming Areas of the Eastern Cape Province, South Africa. World Mycotoxin J..

[34] Raiola A., Tenore G.C., Manyes L., Meca G., Ritieni A. (2015). Risk Analysis of Main Mycotoxins Occurring in Food for Children: An Overview. Food Chem. Toxicol..

[35] Gong Y.Y., Cardwell K., Hounsa A., Egal S., Turner P.C., Hall A.J., Wild C.P. (2002). Dietary Aflatoxin Exposure and Impaired Growth in Young Children from Benin and Togo: Cross Sectional Study. BMJ.

[36] Lombard M.J. (2014). Mycotoxin Exposure and Infant and Young Child Growth in Africa: What Do We Know?. Ann. Nutr. Metab..

[37] Shirima C.P., Kimanya M.E., Routledge M.N., Srey C., Kinabo J.L., Humpf H.-U., Wild C.P., Gong Y.Y. (2014). A Prospective Study of Growth and Biomarkers of Exposure to Aflatoxin and Fumonisin during Early Childhood in Tanzania. Environ. Health Perspect..

[38] Marasas W.F.O., Riley R.T., Hendricks K.A., Stevens V.L., Sadler T.W., Gelineau-van Waes J., Missmer S.A., Cabrera J., Torres O., Gelderblom W.C.A. (2004). Fumonisins Disrupt Sphingolipid Metabolism, Folate Transport, and Neural Tube Development in Embryo Culture and in Vivo: A Potential Risk Factor for Human Neural Tube Defects among Populations Consuming Fumonisin-Contaminated Maize. J. Nutr..

[39] Missmer S.A., Suarez L., Felkner M., Wang E., Merrill A.H., Rothman K.J., Hendricks K.A. (2006). Exposure to Fumonisins and the Occurrence of Neural Tube Defects along the Texas-Mexico Border. Environ. Health Perspect..

[40] Smith L.E., Prendergast A.J., Turner P.C., Mbuya M.N.N., Mutasa K., Kembo G., Stoltzfus R.J. (2015). The Potential Role of Mycotoxins as a Contributor to Stunting in the SHINE Trial. Clin. Infect. Dis..

[41] Spencer P.S., Schmutzhard E., Winkler A.S. (2017). Nodding Syndrome in the Spotlight–Placing Recent Findings in Perspective. Trends Parasitol..

[42] Stacey H.J., Woodhouse L., Welburn S.C., Jones J.D. (2019). Aetiologies and Therapies of Nodding Syndrome: A Systematic Review and Meta-Analysis. J. Glob. Health Rep..

[43] Bernhoft A., Behrens G.H.G., Ingebrigtsen K., Langseth W., Berndt S., Haugen T.B., Grotmol T. (2001). Placental Transfer of the Estrogenic Mycotoxin Zearalenone in Rats. Reprod. Toxicol..

[44] Fukui Y., Hayasaka S., Itoh M., Takeuchi Y. (1992). Development of Neurons and Synapses in Ochratoxin A-Induced Microcephalic Mice: A Quantitative Assessment of Somatosensory Cortex. Neurotoxicol. Teratol..

[45] Partanen H.A., El-Nezami H.S., Leppänen J.M., Myllynen P.K., Woodhouse H.J., Vähäkangas K.H. (2010). Aflatoxin B1 Transfer and Metabolism in Human Placenta. Toxicol. Sci. Off. J. Soc. Toxicol..

[46] Poapolathep A., Sugita-Konishi Y., Phitsanu T., Doi K., Kumagai S. (2004). Placental and Milk Transmission of Trichothecene Mycotoxins, Nivalenol and Fusarenon-X, in Mice. Toxicon.

[47] Ritieni A., Santini A., Mussap M., Ferracane R., Bosco P., Gazzolo D., Galvano F. (2010). Simultaneous Determination of Mycotoxins in Biological Fluids by LC-MS/MS. Front. Biosci. Elite Ed..

[48] Turner P.C., Flannery B., Isitt C., Ali M., Pestka J. (2012). The Role of Biomarkers in Evaluating Human Health Concerns from Fungal Contaminants in Food. Nutr. Res. Rev..

[49] Warth B., Braun D., Ezekiel C.N., Turner P.C., Degen G.H., Marko D. (2016). Biomonitoring of Mycotoxins in Human Breast Milk: Current State and Future Perspectives. Chem. Res. Toxicol..

[50] Zinedine A., Soriano J.M., Moltó J.C., Mañes J. (2007). Review on the Toxicity, Occurrence, Metabolism, Detoxification, Regulations and Intake of Zearalenone: An Oestrogenic Mycotoxin. Food Chem. Toxicol..

[51] Da Rocha M.E.B., da Freire F.C.O., Erlan Feitosa Maia F., Izabel Florindo Guedes M., Rondina D. (2014). Mycotoxins and Their Effects on Human and Animal Health. Food Control.

[52] Williams J.H., Phillips T.D., Jolly P.E., Stiles J.K., Jolly C.M., Aggarwal D. (2004). Human Aflatoxicosis in Developing Countries: A Review of Toxicology, Exposure, Potential Health Consequences, and Interventions. Am. J. Clin. Nutr..

[53] Pestka J.J. (2010). Deoxynivalenol: Mechanisms of Action, Human Exposure, and Toxicological Relevance. Arch. Toxicol..

[54] Yagen B., Joffe A.Z. (1976). Screeing of Toxic Isolates of Fusarium Poae and Fusarium Sporotrichiodes Involved in Causing Alimentary Toxic Aleukia. Appl. Environ. Microbiol..

[55] Duringer J., Craig A., Palmer V., Spencer P., Rahman A., Sandhu P.S. Beauvericin in sorghum proximate to Nodding Syndrome. Proceedings of the 2nd Conference on Chemical Science and Engineering.

[56] Wu S.N., Chen H.Y., Liu Y.C., Chiang H.T. (2002). Block of L-Type Ca^2+^ Current by Beauvericin, a Toxic Cyclopeptide, in the NG108-15 Neuronal Cell Line. Chem. Res. Toxicol..

[57] Ayalew A., Fehrmann H., Lepschy J., Beck R., Abate D. (2006). Natural Occurrence of Mycotoxins in Staple Cereals from Ethiopia. Mycopathologia.

[58] Ediage E.N., Di Mavungu J.D., Song S.Q., Sioen I., De Saeger S. (2013). Multimycotoxin Analysis in Urines to Assess Infant Exposure: A Case Study in Cameroon. Environ. Int..

[59] Hove M., De Boevre M., Lachat C., Jacxsens L., Nyanga L.K., De Saeger S. (2016). Occurrence and Risk Assessment of Mycotoxins in Subsistence Farmed Maize from Zimbabwe. Food Control.

[60] Kitya D., Bbosa G.S., Mulogo E. (2010). Aflatoxin Levels in Common Foods of South Western Uganda: A Risk Factor to Hepatocellular Carcinoma. Eur. J. Cancer Care.

[61] Shephard G.S., Burger H.-M., Gambacorta L., Gong Y.Y., Krska R., Rheeder J.P., Solfrizzo M., Srey C., Sulyok M., Visconti A. (2013). Multiple Mycotoxin Exposure Determined by Urinary Biomarkers in Rural Subsistence Farmers in the Former Transkei, South Africa. Food Chem. Toxicol..

[62] Ezekiel C.N., Sulyok M., Ogara I.M., Abia W.A., Warth B., Šarkanj B., Turner P.C., Krska R. (2019). Mycotoxins in Uncooked and Plate-Ready Household Food from Rural Northern Nigeria. Food Chem. Toxicol..

[63] Pfohl-Leszkowicz A., Manderville R. (2007). Ochratoxin A: An Overview on Toxicity and Carcinogenicity in Animals and Humans. Mol. Nutr. Food Res..

[64] Abia W.A., Warth B., Sulyok M., Krska R., Tchana A., Njobeh P.B., Turner P.C., Kouanfack C., Eyongetah M., Dutton M. (2013). Bio-Monitoring of Mycotoxin Exposure in Cameroon Using a Urinary Multi-Biomarker Approach. Food Chem. Toxicol..

[65] Šarkanj B., Warth B., Uhlig S., Abia W.A., Sulyok M., Klapec T., Krska R., Banjari I. (2013). Urinary Analysis Reveals High Deoxynivalenol Exposure in Pregnant Women from Croatia. Food Chem. Toxicol..

[66] Wallin S., Gambacorta L., Kotova N., Warensjö Lemming E., Nälsén C., Solfrizzo M., Olsen M. (2015). Biomonitoring of Concurrent Mycotoxin Exposure among Adults in Sweden through Urinary Multi-Biomarker Analysis. Food Chem. Toxicol..

[67] Lahouar A., Crespo-Sempere A., Marín S., Saïd S., Sanchis V. (2015). Toxigenic Molds in Tunisian and Egyptian Sorghum for Human Consumption. J. Stored Prod. Res..

[68] Domijan A.M. (2012). Fumonisin B(1): A Neurotoxic Mycotoxin. Arch. Ind. Hyg. Toxicol..

[69] Frederickson D.E., Mantle P.G., Milliano W.A.J.D. (1991). *Claviceps Africana* Sp. Nov.; The Distinctive Ergot Pathogen of Sorghum in Africa. Mycol. Res..

[70] Blaney B.J., McLennan S.R., Kidd J.F., Connell J.A., McKenzie R.A., Downing J.A. (2011). Effect of Sorghum Ergot (*Claviceps Africana*) on the Performance of Steers (*Bos Taurus*) in a Feedlot. Anim. Prod. Sci..

[71] Blaney B.J., McKenzie R.A., Walters J.R., Taylor L.F., Bewg W.S., Ryley M.J., Maryam R. (2000). Sorghum Ergot (*Claviceps Africana*) Associated with Agalactia and Feed Refusal in Pigs and Dairy Cattle. Aust. Vet. J..

[72] Ohashi H., Ueno A., Nakao T., Ito J., Kimura K., Ishikawa M., Kawai H., Iijima H., Osawa T. (1999). Effects of Ortho-Substituent Groups of Sulochrin on Inhibitory Activity to Eosinophil Degranulation. Bioorg. Med. Chem. Lett..

[73] Ohashi H., Motegi Y., Kita H., Gleich G.J., Miura T., Ishikawa M., Kawai H., Fukamachi H. (1998). Sulochrin Inhibits Eosinophil Activation and Chemotaxis. Inflamm. Res..

[74] Qian Z.-J., Zhang C., Li Y.-X., Je J.-Y., Kim S.-K., Jung W.-K. (2011). Protective Effects of Emodin and Chrysophanol Isolated from Marine Fungus *Aspergillus* sp. on Ethanol-Induced Toxicity in HepG2/CYP2E1 Cells. Evid. Based Complement. Altern. Med. ECAM.

[75] Dong X., Fu J., Yin X., Cao S., Li X., Lin L., Huyiligeqi Ni J. (2016). Emodin: A Review of Its Pharmacology, Toxicity and Pharmacokinetics. Phytother. Res..

[76] Warth B., Sulyok M., Krska R. (2013). LC-MS/MS-Based Multibiomarker Approaches for the Assessment of Human Exposure to Mycotoxins. Anal. Bioanal. Chem..

[77] Šarkanj B., Ezekiel C.N., Turner P.C., Abia W.A., Rychlik M., Krska R., Sulyok M., Warth B. (2018). Ultra-Sensitive, Stable Isotope Assisted Quantification of Multiple Urinary Mycotoxin Exposure Biomarkers. Anal. Chim. Acta.

[78] Schwartz-Zimmermann H.E., Hametner C., Nagl V., Fiby I., Macheiner L., Winkler J., Dänicke S., Clark E., Pestka J.J., Berthiller F. (2017). Glucuronidation of Deoxynivalenol (DON) by Different Animal Species: Identification of Iso-DON Glucuronides and Iso-Deepoxy-DON Glucuronides as Novel DON Metabolites in Pigs, Rats, Mice, and Cows. Arch. Toxicol..

[79] Warth B., Sulyok M., Fruhmann P., Mikula H., Berthiller F., Schuhmacher R., Hametner C., Abia W.A., Adam G., Fröhlich J. (2012). Development and Validation of a Rapid Multi-Biomarker Liquid Chromatography/Tandem Mass Spectrometry Method to Assess Human Exposure to Mycotoxins. Rapid Commun. Mass Spectrom. RCM.

[80] United States Food and Drug Administration Guidance for Industry Bioanalytical Method Validation. http://www.fda.gov/downloads/Drugs/Guidances/ucm070107.pdf.

