# Supplementary material for: Case-Control Study of Nodding Syndrome in Acholiland: Urinary Multi-Mycotoxin Screening
Source: Toxins (Basel). 2021 Apr 27;13(5):313. doi: 10.3390/toxins13050313 (PMC8145943; doi:10.3390/toxins13050313)
Supplement: Supplementary file 1 [file toxins-13-00313-s001.zip › toxins-1165115-supplementary DOI.pdf]

Supplementary Materials: Case-Control Study of Nodding Syndrome in Acholiland: Uri-nary Multi-Mycotoxin Screening

Jennifer Duringer, Rajarshi Mazumder, Valerie Palmer, A. Morrie Craig and Peter Spencer *

Table S1. Validation data

Compound	Linearity	Calibration curve range	LOD (ng/mL)	LOQ (ng/mL)
Alpha-zearalenol	0.995	5–500 ng/mL	5	20
Beta-zearalenol	0.995	20–500 ng/mL	20	20
Aflatoxin M1	0.995	1–50 ng/mL	1	5
Fumonisin B1	0.996	0.1–50 ng/mL	0.1	2.5
Fumonisin B2	0.993	0.5–50 ng/mL	0.5	2.5
HT-2 toxin	0.893	0.2–5 ng/mL	0.2	5
Ochratoxin A	0.990	0.5–100 ng/mL	0.5	0.5
T-2 toxin	0.967	1–20 ng/mL	1	10
Zearalenone	0.997	10–500 ng/mL	10	10

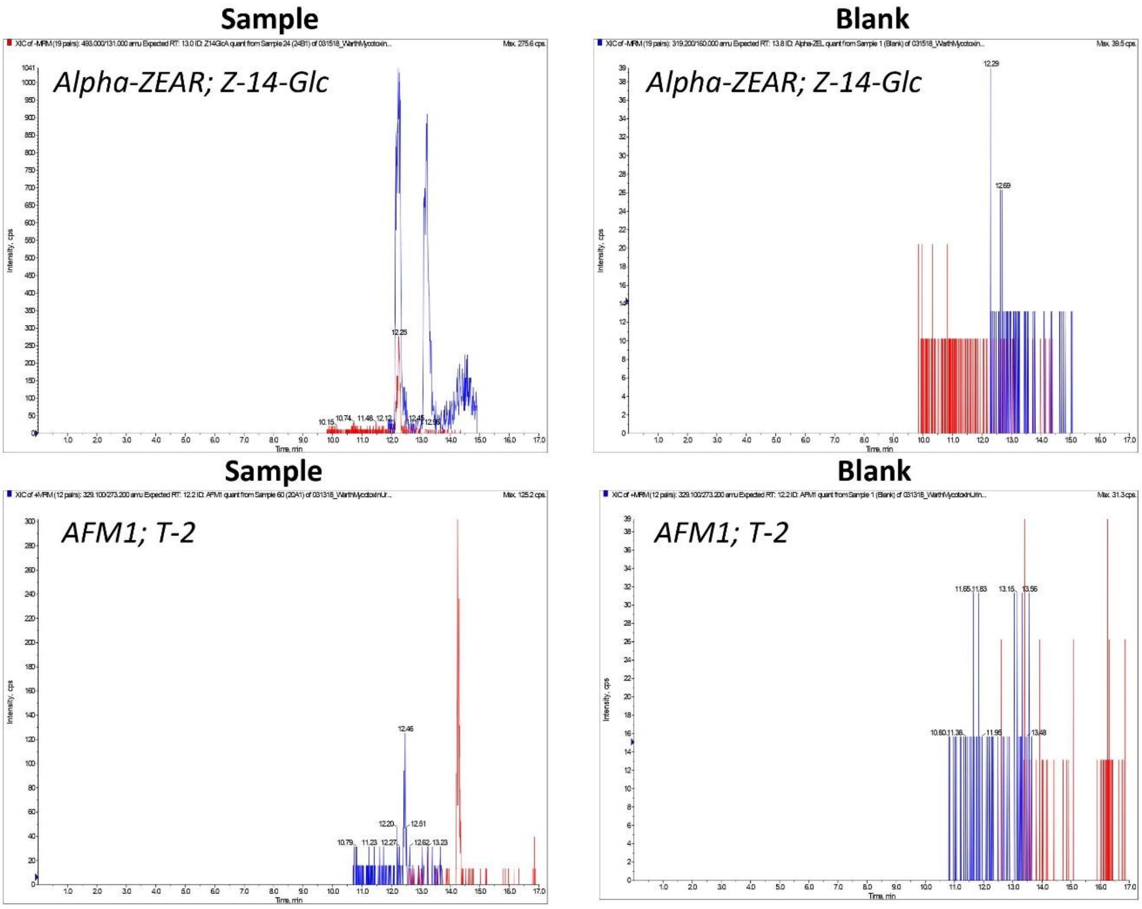

Figure S1. Extracted ion chromatograms of mycotoxins detected in urine of Acholi children, as compared to method blank (1:9 ACN:water).
